# Smartphones in mental health: a critical review of background issues, current status and future concerns

**DOI:** 10.1186/s40345-019-0164-x

**Published:** 2020-01-10

**Authors:** Michael Bauer, Tasha Glenn, John Geddes, Michael Gitlin, Paul Grof, Lars V. Kessing, Scott Monteith, Maria Faurholt-Jepsen, Emanuel Severus, Peter C. Whybrow

**Affiliations:** 1 Department of Psychiatry and Psychotherapy, University Hospital Carl Gustav Carus, Medical Faculty, Technische Universität Dresden, Fetscherstr. 74, 01307 Dresden, Germany; 2 ChronoRecord Association, Fullerton, CA USA; 3 Department of Psychiatry, University of Oxford, Warneford Hospital, Oxford, UK; 4 Department of Psychiatry and Biobehavioral Sciences, Semel Institute for Neuroscience and Human Behavior, University of California Los Angeles (UCLA), Los Angeles, CA USA; 5 Mood Disorders Center of Ottawa, Ottawa, Canada; 6 Department of Psychiatry, University of Toronto, Toronto, ON Canada; 7 Copenhagen Affective Disorder Research Center (CADIC), Psychiatric Center Copenhagen, Rigshospitalet, Copenhagen, Denmark; 8 Michigan State University College of Human Medicine, Traverse City Campus, Traverse City, MI USA

**Keywords:** Smartphone, Cellphone, Technology, Mental illness, Psychiatry, Wearables

## Abstract

There has been increasing interest in the use of smartphone applications (apps) and other consumer technology in mental health care for a number of years. However, the vision of data from apps seamlessly returned to, and integrated in, the electronic medical record (EMR) to assist both psychiatrists and patients has not been widely achieved, due in part to complex issues involved in the use of smartphone and other consumer technology in psychiatry. These issues include consumer technology usage, clinical utility, commercialization, and evolving consumer technology. Technological, legal and commercial issues, as well as medical issues, will determine the role of consumer technology in psychiatry. Recommendations for a more productive direction for the use of consumer technology in psychiatry are provided.

## Background

A smartphone is a transformational technology. The many benefits include instant communications and access to information from anywhere while using a simple, graphical, finger-based interface. One smartphone eliminates the need to carry many devices including a phone, camera, speakers, WiFi adapter, and a GPS system. A smartphone allows the user to download and run applications (apps). The built-in sensors can provide measurements and contextual information, and by integrating communications into an app, the user needs few connectivity skills. In 2012, a consumer smartphone had more than 100 times the computing power of the average satellite (NASA 2012).

Many thought the use of a smartphone in psychiatry would enable new measures of patient mental state and behavior to assist with patient screening, diagnosis, monitoring and treatment (Glenn and Monteith 2014a; Mohr et al. 2017; Luxton et al. 2011), such as for bipolar disorder (Faurholt-Jepsen et al. 2014, 2018; Harrison et al. 2016). The future is often envisioned in which actionable data from apps, both recommended by psychiatrists and selected by patients, are seamlessly returned to the EMR, and data from the apps would provide clinically useful measures to the physician and immediate feedback to assist patients. Although there are many mental health apps available for smartphones, their expected value has not been realized. This paper will discuss some of the complex reasons why the smartphone has not reached this potential for collecting patient data in psychiatry. Consumer technology usage, clinical utility, commercialization, and evolving consumer technology will be discussed, followed by recommendations to enhance the use of consumer technology in psychiatry.

## Consumer technology usage

### Smartphone use is not universal

Smartphone use varies throughout the world. From a global perspective, in 2017 there were 5 billion unique mobile subscribers with 57% of connections using a smartphone (GSMA 2018a). For the 5 billion mobile subscribers, the top 3 uses of a cellphone are to make or receive cellular calls, send or receive text messages (SMS), and use messaging apps, while downloading and using apps for purposes other than messaging is ranked number 9 (GSMA 2018b). In 2018, 95% of the US population owned a cellphone, and 77% of the cellphones were smartphones (Pew Research 2018). The percentage of cellphones that are smartphones in the US has remained constant since 2016 (Pew Research 2018).

Worldwide, smartphone use is unevenly distributed across the population. Older people use a smartphone significantly less often than younger people (Berenguer et al. 2017). In the US, about 40% of community dwelling adults age 65 years or older own a smartphone (Anderson and Perrin 2017) with barriers to use including visual and physical impairments (Kuerbis et al. 2017; Bauer et al. 2018a). Additionally, older people often use a smartphone as a standard feature phone, making calls but never downloading apps (Berenguer et al. 2017). People with low income may only have intermittent access to smartphones (Gonzales 2016). Smartphone use by those with serious mental illness is generally lower than for the general population (Klee et al. 2016; Glick et al. 2016; Abu Rahal et al. 2018; Carpenter-Song et al. 2018). Smartphone use is also associated with more education (Pew Research 2018).

### Alternatives to smartphones

Consumers own many technologies in addition to a smartphone. In the US, in 2018, 73% of the population owned a desktop or laptop, and 53% owned a tablet (Pew Research 2018). Most owners of desktops or laptop also own peripheral devices such as printers and scanners. People use smartphones and desktops/laptops for different daily activities, varying by age (Bröhl et al. 2018). Although younger generations use a smartphone most frequently to perform daily activities, all age groups in an international sample preferred to use a desktop PC/laptop for writing emails or letters, and for passing on confidential information (Bröhl et al. 2018). In an international sample of patients with bipolar disorder, 81% looked for information on the Internet, with 89% of these preferring to search from a desktop/laptop (Conell et al. 2016).

### Wearables

In 2018, about 20% of adult Americans used wearable technology at least once a month (Statistica 2019c). Wearable technology refers to accessories and clothing incorporating computer technologies, including smart watches, fitness trackers, smart clothing, and ear-worn devices (Godfrey et al. 2018). Smart watches and fitness trackers are the most popular wearables (Gartner 2018), and sales are growing rapidly (IDC 2019). Wearables are playing an increasing role in healthcare (Amft 2018), including mental health (Behar et al. 2019). Many smartphone apps connect to sensors in wearable technologies (research2Guidance 2018). For example, Abilify MyCite are aripiprazole pills with an embedded sensor that communicates to a wearable patch when ingested, which then transmits data to a smartphone app (FDA 2017a).

### Medical apps from the app stores

The app stores offer trusted one-stop shopping for consumers to obtain software for their smartphone’s operating system, few entry barriers for app developers, and instant credibility for small developers (Deloitte 2018). Apple and Google dominate the distribution of apps from their stores. In 2018, there were 2.1 million apps in the Android store and 2 million apps in the Apple store (Statistica 2019a). The number of health apps and health app publishers keeps growing. In 2017, there were 325,000 health apps available from 84,000 health app publishers, with about 78,000 apps added in the last year (research2guidance 2018). The majority of apps are developed by technology companies or app developers outside of the healthcare industry (research2guidance 2016; Ahmed et al. 2018). Most health apps have less than 5000 yearly downloads (research2guidance 2017). The selection of mental health apps available in the app stores is constantly changing. In a 9-month study of apps available in Australian Google Play and Apple iTunes stores related to depression, bipolar disorder and suicide, 50% of results changed after 4 months with an app being removed every 2.9 days (Larsen et al. 2016b).

### App retention rates

The user retention rate for smartphone apps in the general population is low. About 25% of users abandon apps after one use (Rodde 2019). For both Android and Apple smartphones, the app retention rate worldwide after 90 days was just 4% in 2016 (Statistica 2019b). Even the wildly popular Pokémon Go app dropped 80% of users in a few months (comScore 2017a).

Similarly, reports for health apps, including mental health related, show limited downloads and poor retention, especially outside of clinical trials and research settings. In national surveys in Germany, 20.5% of adult smartphone users 35 years or older, and 16.5% older adults used a health app (Ernsting et al. 2017; Rasche et al. 2018). In a national survey of smartphone users in the US, 58% had downloaded a health app but about half had stopped using it (Krebs and Duncan 2015). In other studies using national data, people who used health apps were younger, richer and in excellent health (Carroll et al. 2017), and a heath app was downloaded by 12% of those with depression (Robbins et al. 2017). In studies of patients with mental illness, a mental health app was downloaded by 10.7% at a VA facility (Lipschiz et al. 2019). About 10% of patients both at a state clinic and at a private insurance clinic were using a mental health app (Torous et al. 2018). A review of digital self-help apps or programs for depression and anxiety, involving 8 to 40,000 downloads or registrations per month, reported 21–88% using at least once, and 0.5–28.6% continuing after 6 weeks (Fleming et al. 2018). Only 18.7% of a US Hispanic/Latino population enrolled in a depression clinical trial downloaded the treatment app (Pratap et al. 2018).

Selective use and low retention rates directly affects research. Data collected from apps or social media are generally not representative of the national population, people with mental illness, or of people with a specific condition (Monteith and Glenn 2016). Additionally, with the low retention rates, researchers using a dataset from the same app or social media platform at different times may be investigating different user populations (Althoff 2017).

While some patients will use and benefit from mental health apps, all consumer technologies have selective use and low retention rates. High interest in mental health apps will not automatically lead to high use (Torous et al. 2018). Mental health app retention may be improved by using the app in clinical settings and providing a free smartphone and data plan (Faurholt-Jepsen et al. 2015, 2019; Achtyes et al. 2019). Given that only about 50% of patients take medications as prescribed (Brown and Bussell 2011), expectations for the use of recommended medical apps should not be overly optimistic.

### Selection of mental health apps

Most consumers find mental health apps through social media, web searches or word of mouth rather than by professional recommendation (Schueller et al. 2018). Smartphone owners often rely on online app reviews but these can be misleading. For example, none of the 25 most popular iPhone apps for anxiety and worry, as indexed by user ratings, included content consistent with evidence-based treatments (Kertz et al. 2017). Users gave high reviews to a very popular but inaccurate blood pressure app that was withdrawn from the market (Plante et al. 2018). Patients and physicians may have a different perspective on the quality or usability of apps (Singh et al. 2016, 2019), including for bipolar disorder (Nicholas et al. 2015). In a study of top ranked mental health apps from Google Play and iTunes stores, scientific language, not direct evidence, was the most frequently employed strategy to support claims of effectiveness (Larsen et al. 2019). Additionally, there are many services for unscrupulous app developers to game the app review process, such as by paying users to write reviews (Hill 2018).

### Attitudes towards apps and wearables

People have varied attitudes towards the use of apps and wearables. Many people with chronic medical illness reject self-monitoring, finding it annoying, depressing, a burden, or prefer to forget they are ill (Lupton 2013; Bauer et al. 2017). The very process of measurement may hinder enjoyment of physical activities and decrease well-being (Toner 2018; Etkin 2016). Some may fixate on one measure, such as a step count, and ignore other health related issues (Felde 2019). Patients with mental illness may prefer apps that support relaxation and time management, rather than apps that directly target their disorders (Dragovic et al. 2018). Some feel that the privacy risks associated with wearable devices pose a threat to their health and well-being (Marakhimov and Joo 2017). Some feel they are being increasingly asked to rely on their own observations and online findings, and view this as being outside the traditional healthcare system (Vesnic-Alujevic et al. 2018). Many of the patients who do not use mental health apps may prefer direct, personal contact with a psychiatrist.

## Clinical utility

### Issues with regulation of apps and wearables

In the US, very few medical apps and wearables, including for mental health, require FDA review. Regulation is limited primarily to software that is an accessory to a regulated medical device, or that will transform a mobile platform into a regulated medical device (Davis 2017; FTC 2016). The vast majority of medical apps that pose “minimal risk” to a user are outside of FDA enforcement. For example, mental health apps that help to self-manage but do not claim to diagnose or provide specific treatment suggestions would pose “minimal risk” (FTC 2016; Armontrout et al. 2018). Even with software devices that require FDA review, under the FDA Digital Health Software Pre-certification Program, the manufacturer, not the actual software product, is certified based on a company culture of software quality and commitment to patient safety (Terry 2019). Although post-market surveillance is required, consumers and physicians may not realize that FDA pre-certification does not necessarily mean that products were proven safe and effective before release (Lee and Kesselheim 2018). An unforeseen result of this regulatory policy may be the release of consumer products deemed controversial by physicians, such as the use of Apple Watch to detect undiagnosed atrial fibrillation (Mandrola and Foy 2019; Packer 2019; Rowland 2018). Lack of regulation of medical and mental health apps is a growing international problem (Parker et al. 2019).

### Measurement accuracy of apps and wearables

Sensors in smartphones and wearables offer the potential for physiological measurement and remote monitoring (Lowe and Ólaighin 2014). However, measurement inaccuracies are frequently reported including overestimation, underestimation, high variability, misclassification of results, and lack of agreement with gold standards (Hwang 2018). See Tables 1 and 2. Frequent errors are related to properties of the embedded sensors and smartphone hardware, the medical apps, and the human use of smartphones and wearables.

**Table 1:** Accuracy problems in studies using physiological measurements by smartphone apps

Measure	App	Smartphone	Participants	Finding	Study
Blood pressure	Instant Blood Pressure	iPhone 5 and 6.	85 patients and staff; 53% with hypertension.	Measures “were highly inaccurate”	Plante et al. (2016)
Heart rate	Instant Heart Rate Heart Fitness Whats My Heart Rate Cardio Version	iPhone 4 and 5.	108 patients, exluding those in critical condition.	“substantial performance differences” between the four apps	Coppetti et al. (2017)
Heart rate Blood pressure Oxygen saturation	Instant Blood Pressure Instant Blood Pressure Pro Pulse Oximeter Pulse oximeter Pro	iPhone 5S	100 healthy participants	“applications evaluated do not provide clinically meaningful data” “inaccurate data.. can potentially contribute to patient harm”	Alexander et al. (2017)
Heart rate	Runtastic Heart Rate Monitor Instant heart rate+	iPhone	15 regularly active college students	“Poor correlation to ECG” during moderate to high intensity exercise	Bouts et al. (2018)
Step counting	Argus: calorie counter and step	Android phones: Samsung, OnePlus, Moto, Oppo, Galazy, Huawei, LG, Google, Sony and Agora running Android 4.4 to 8.1 Apple: iPhone 6, 6S, 7, 8, running iOS10.3–11.4.	48 healthy participants	“extraordinarily large error ranges for both..phones” “appear unsuitable to detect steps in short, slow, or non-stereotypical gait patterns”	Brodie et al. (2018)
Sleep	Sleep time	iPhone 4 s and 5	20 participants with no sleep disorder	“absolute parameters and sleep staging…. correlate poorly with polysomnography”	Bhat et al. (2015)
Sleep	MotionX 24/7	iPhone 4	78 children and adolescents with suspected sleep disordered breathing	“did not accurately reflect sleep or wake and should be used with caution”	Toon et al. (2016)

**Table 2:** Accuracy problems in studies using physiological measurements by wearables

Measure	Number tested	Wearables	Patients	Finding	Study
Total energy expenditure	12 devices	Withings Pulse Jawbone Garmin Vivofit Suzuken Lifecorder EX Panasonic Actimaker Epson Pulsense Tanita-AM-160 Fitbit Flex Misfit Shine Omron Active Style Pro Omron CaloriScan	19 healthy adults, not obese.	“absolute values differed widely among products and varied significantly from the gold standard measures”	Murakami et al. (2016)
Step count	3 pedometers	Yamax Digiwalker Fitbit	14 young health participants walking at 3 speeds	“all the evaluated devices had high error rates at 1 km/h” (slow walking speeds).	Beevi et al. (2016)
Step count	10 activity trackers	Polar Loop Garmin Vivosmart Fitbit Charge HR Apple Watch Sport Pebble Smartwatch Samsung Gear S Misfit Flash Jawbone Up Move Flyfit Moves	31 healthy participants on a treadmill	“Test–retest validity depends on walking speed”; “consumer activity trackers perform better at average and vigorous walking speed…”	Fokkema et al. (2017)
Sleep	2 monitors	Withings Pulse Jawbone Up	36, including 22 with obstructive sleep apnea (OSA)	“confirmed… in patients suffering from OSA, the limited performance of wearable sleep monitors”	Gruwez et al. (2019)
Sleep	2 monitors	Fitbit Change 2 Neuroon	25 students	Underestimate light sleep and overestimate deep sleep. “Reasonably satisfactory for general purpose and non-clinical use”	Liang and Martell (2018)

The sensors embedded in smartphones and other consumer devices are generally not professional grade due to manufacturing costs and power requirements, and may be inaccurate (Grewal and Andrews 2010; Puentes et al. 2013; del Rosario et al. 2015; Grammenos et al. 2018). For example, there is sensor bias (defined as the average sensor output at zero sensor input) in the accelerometer and gyroscope, as measured in 61 smartphones including models from Samsung, Apple, Huawei, and Sony (Kos et al. 2016). This bias must be calibrated and compensated for by sensor type and model, especially in apps designed to run across multiple smartphone devices. Additionally, when the same sensor is embedded in a smartphone from a different manufacturer using a different operating system, results may differ since sensor signals are processed by the operating system before being presented to the apps. Apps from different developers, which are based on the same sensor and run on the same device, may also provide different results due to different software programming techniques.

The sensors that are embedded in smartphones often change over time, which may lead to varying measurement results. From a manufacturing perspective, all components of a smartphone including sensors may have cost or power saving revisions through a variety of approaches including part substitution (using a different part with identical or similar form, fit and function), and by redesign (upgrading the system to utilize newer parts) (Solomon et al. 2000). As a smartphone goes through its product life cycle, it is very likely that components, including sensors, for the same model have been revised, which may change measured results. Additionally, specific models of smartphones have a short lifespan with new models often introduced yearly. When smartphone models change, it is very likely that hardware components including sensors will change and software must be recalibrated (Li et al. 2010; del Rosario et al. 2015; Kos et al. 2016). The sensors used in wearables also change frequently and will result in inconsistent data collection (Amft and Van Laerhoven 2017).

Hardware features of the smartphone itself can influence measurements including power consumption, processor speed, smartphone size, the position of sensor in the device, and sensor ability to handle noisy indoor and outdoor environmental conditions (Parpinel et al. 2017; Agu et al. 2013; GPS 2017). Individual actions also may impact measurement accuracy such as where and how a smartphone is held (Agu et al. 2013; Vezočnik and Juric 2019), and not following instructions on how to use an app. Patients may forget to charge a smartphone, stop using sensor based apps to preserve battery life, turn off the smartphone, be out of range for data transmission, or loan the phone to another person (Agu et al. 2013; Boonstra et al. 2018). Similar problems also occur with wearables. A review of 67 studies of Fitbit devices found that other than measures of steps in adults without mobility limitations, the device is unlikely to provide sufficiently accurate measurement for clinical medicine or research (Feehan et al. 2018).

The various revisions of sensors and models for the smartphone and other devices are of major concern since the vast majority of apps are not regulated by the FDA as a medical device. A traditional medical device would require testing, validation and recertification by a regulatory body after a hardware or software change that may be critical to safety or efficacy, before the modified device is released to the public (FDA 2017b).

### Blue light exposure

Measurement using smartphone apps and small devices is also a concern because light-emitting diode (LED) backlights are used to enhance daytime brightness and contrast of displays (Gringras et al. 2015; Oh et al. 2015). Unlike conventional lighting, LEDs emit bright blue light at a wavelength close to the peak sensitivity for non-visual circadian photoreception (Gringras et al. 2015; Oh et al. 2015). In studies primarily of healthy volunteers, exposure to blue light may disrupt a variety of circadian functions (Tosini et al. 2016; Bauer et al. 2018b), even at low intensities (Prayag et al. 2019). For example, even “night-time” settings on devices may emit light far above the predicted threshold for melatonin suppression (Prayag et al. 2019). When a smartphone is used as a measuring device, exposure to blue light can directly impact the patient and influence the measurement of a wide range of variables including alertness, cognition, sleep, and activity levels. Without understanding if exposure to blue light from smartphones, other devices, or ambient lighting is impacting what is being measured, data collected are difficult to interpret (Bauer et al. 2018b).

### Efficacy not proven

A lack of efficacy of smartphone apps and wearables extends throughout medicine. A Cochrane review of automated telephone communication systems, often smartphone based, included 132 clinical trials and over 4 million participants across specialties (Posadzki et al. 2016). Positive effects were found from reminders, including increased prevention screenings and appointment attendance, while other effects varied by condition with “little or no effect” in mental health (Posadzki et al. 2016; Foster and Callans 2017). Table 3 summarizes systematic reviews of apps related to mental health that are available to consumers. While many studies discuss the potential of mental health apps, there is little clinically validated evidence. For example, a review of 100 studies that used a mental health app for a wide range of conditions, only 14 had clinically validated evidence (Wang et al. 2018). Additionally, reviews that focus on the mental health apps with controlled trials generally have conclusions such as promising but little evidence today, studies of mixed quality, and more, larger trials needed (Dogan et al. 2017; Byambasuren et al. 2018; Firth and Torous 2015; Wang et al. 2018). However, given the low cost of entry for app developers, the vast majority will never be able to afford even a simple clinical trial to establish efficacy (Foster and Callans 2017). This is a major challenge: how do we test the effectiveness of these applications in a timely, robust and cost-effective manner? Are clinical trials always necessary? Answering these questions is critical and will need considerable methodological innovation.

**Table 3:** Review articles on quality and effectiveness of apps in English related to mental health

Category	Source	Country of search	Dates of search	Number apps from search	Number apps evaluated, downloaded, included	Conclusion	Study
Medication adherence	Google Play, Apple App store	UK	Apr 2015	5888	805 evaluated; 681 included; 420 free downloaded	“the vast majority of current adherence app offerings on repositories lack any evidence base of effectiveness”	Ahmed et al. (2018)
Prevent driving after drinking	Google Play, Apple App store	Australia	Jun 2015	2907	70 evaluated; 58 included.	“Most apps for drink driving prevention are not engaging, and none have as yet been tested in trials to determine their effectiveness in reducing drink driving behavior”	Wilson et al. (2016)
Suicide prevention	Google Play, Apple iTunes store	Australia	Unspecified	1271	856 evaluated 123 downloaded; 49 included.	“Clinicians should be wary in recommending apps, especially as potentially harmful content can be presented as helpful”	Larsen et al. (2016a)
Crystal methamphetamine	Google Play, Apple iTunes store	Australia	Apr 2017	2205	1983 evaluated; 30 downloaded; 18 included.	“This study demonstrates a shortage of high-quality educational and engaging smartphone apps specifically related to methamphetamine”	Chapman et al. (2018)
Sleep self-management	Google Play, Apple iTunes store, Amazon Appstore, Microsoft Appstore	US	Apr 2017	2431	1981 evaluated; 148 downloaded; 73 included.	“few apps meet prespecified criteria for quality, content, and functionality for sleep self-management. Despite the rapid evolution of sleep self-management apps, lack of validation studies is a significant concern that limits the clinical value of these apps”	Choi et al. (2018)
Bipolar Disorder	Google Play, Apple App store	Australia	Jul 2014	571	438 evaluated; 82 included.	“In general, the content of currently available apps for BD is not in line with practice guidelines or established self-management principles”	Nicholas et al. (2015)
Depression - CBT or behavioral activation apps	Google Play, Apple App store, literature	Canada	Nov 2015	310	117 evaluated; 12 included.	“Despite the growing public demand, there is a concerning lack of appropriate CBT or BA apps, especially from a clinical and legal point of view”	Huguet et al. (2016)
Attention and cognitive bias modification apps	Google Play, Apple iTunes store	Singapore	Sept, 2017	98	17 included.	“only 1 commercial app had been evaluated in the published literature”	Zhang et al. (2018)
Physical activity	Google Play, Apple iTunes store	UK	Oct, 2016	400	156 evaluated; 65 included.	“..there were substantial shortcomings in the areas of data safety and likelihood of effectiveness of the apps assessed”	Bondaronek et al. (2018)
Social anxiety	Google Play, Apple iTunes, Windows store	New Zealand	June, 2016	1154	73 identified 38 randomly included.	“none of the apps identified have had studies on their effectiveness published”	Alyami et al. (2017)
Anxiety	Google Play, Apple App store	European app stores	Jan, 2017	5078	4404 evaluated; 73 downloaded; 52 included.	“there is a marked discrepancy between the wealth of commercially available apps, and the paucity of data regarding their efficacy and effectiveness”	Sucala et al. (2017)
Smoking cessation for those with psychosis	Google Play, Apple iTunes store	US	July, 2013	766	100 evaluated; 73 included, 9 further analyzed.	“ongoing poor quality of most apps”. “smoking cessation apps may be inaccessible or ineffective for most smokers with psychotic disorders”	Ferron et al. (2017)
Weight management	Google Play, Apple iTunes store	Australia	Aug, 2014	800 most popular	55 evaluated; 28 included.	“Overall, the most popular commercial apps for weight management are suboptimal in quality, given the inadequate scientific coverage and accuracy of weight-related information…”	Chen et al. (2015)
Insomnia	Google Play, Apple iTunes store	US	Nov, 2016	355	12 included.	“despite the hundreds of apps available,…few are adherent to the evidence-based skills and strategies that have been shown effective in managing insomnia”	Yu et al. (2019)

### Passive monitoring

Some apps focus primarily on using a smartphone for ongoing passive monitoring of individuals with mental illness. Passive monitoring collects data from patients without requiring direct patient input, often using only sensors to measure a wide range of variables such as activity level, mobility, physiological measures, speech patterns, and signals of social interactions (Abdullah and Choudhury 2018). Reviews of passive monitoring report similar conclusions, that results are promising but many methodological and interpretive challenges remain, larger trials are needed, and evidence in clinical settings is lacking (Faurholt-Jepsen et al. 2018; Cornet and Holden 2018; Seppälä et al. 2019; Rohani et al. 2018; Goodday and Cipriani 2019). In addition to the hardware and software concerns, there are special concerns with passive monitoring related to stigma and privacy (Bauer et al. 2017). Of patients interested in apps, a significant minority does not want to be monitored and tracked or provide private sensor-based data (Thornton and Kay-Lambkin 2018; Klasnja et al. 2009; Torous et al. 2018; Ben-Zeev et al. 2016; Di Matteo et al. 2018; Hendrikoff et al. 2019). In a study of mobile sensing of 126 adults with depression recruited from the general public in Switzerland, half uninstalled the app within 2 weeks (Wahle et al. 2016). Mood assessment by passive monitoring was not useful at the population level (Pratap et al. 2019). Patient adherence with wearables is also a major problem (Amft 2018).

### Ethical issues with mental health apps

There are many ethical issues associated with mental health apps. Apps are being widely promoted, often containing incorrect information and with unproven efficacy. See Tables 1, 2 and 3. Mental health apps may promote unsafe and misleading messages. For example, potentially harmful information was noted in apps about bipolar disorder (Nicholas et al. 2015), suicide (Larsen et al. 2016a) and alcohol use (Crane et al. 2015). A study of 64 frequently used mental health apps noted two recurring themes: that fragile mental health is ubiquitous, and that individuals can easily manage mental health problems with apps (Parker et al. 2018). These messages may medicalize normal mental states, and be dangerous for those with diagnosed mental illness who need a clear understanding of when to seek professional help (Parker et al. 2018). Problematic use of smartphones is associated with depression and anxiety, and mental health apps may not be appropriate (Elhai et al. 2017; Kim et al. 2015). Lax regulation may allow direct-to-consumer psychotherapy apps to connect users to minimally trained, nonprofessional counselors (Martinez-Martin and Kreitmai 2018). Apps that connect patients to chatbots may provide incomplete responses to simple questions, have limited capacity to recreate human interactions or offer tailored treatment, and may not provide real time access to mental health services when needed (Kretzschmar et al. 2019; Miner et al. 2016).

Another issue relates to recommending apps to patients without sufficient understanding of the patient’s technical competence, awareness of privacy issues, and ability to avoid online harm (Bauer et al. 2017; Torous and Roberts 2017; Gangadharan 2017). Poor digital skills, lack of knowledge of online safety practices, individual traits associated with mental illness, and cognitive impairment all increase vulnerability to online fraud (Monteith and Glenn 2016; Gangadharan 2017; Sheng et al. 2010; Bauer et al. 2017, 2018a).

## Commercialization

### Commercial firms and the digital economy

As commercial firms play an increasing role in providing apps, wearables, and algorithms in medicine, it is important to recognize that the digital economy is based on collecting and selling personal data (Bauer et al. 2017). The dominant business model depends on violating privacy (Narayanan 2018) as commercial firms routinely track all individual online activities and habits (Glenn and Monteith 2014a; Monteith and Glenn 2016). Data are collected, combined with other data, and re-sold as data products. This commodification of consumer data includes data from medical apps and wearables, facial recognition, as well as biometrics used in authentication schemes (Elvy 2018; Roberts 2019). With this distributed and redundant data economy, one should assume that data cannot be permanently deleted (PCAST 2014).

Commercial, academic and governmental organizations purchase data, combine data with other data from all aspects of daily life, and create algorithms that are routinely used to classify people (Bauer et al. 2017; Monteith and Glenn 2016). The use of these classifications extends far beyond behavioral or personalized advertising, and sales of products “recommended for you” (Beales 2010). These algorithm classifications directly impact almost every aspect of daily life, including education, employment, credit, healthcare, criminal justice, and government services (Monteith and Glenn 2016; Bauer et al. 2017). In the US, the market value of personal data was estimated at $76 billion in 2018, increasing 44.9% from 2016 to 2018 (Shapiro and Aneja 2019). Most commercial algorithms are proprietary and opaque, with this lack of transparency posing a variety of safety dangers (Bauer et al. 2019; ACM 2017; AI HLEG 2019). Despite the size, the big data basis for the classification may not be representative of the general population, or of those with mental illness (Monteith et al. 2016). Algorithmic decision-making based on big data may be incorrect, reflect human biases, and incorporate and perpetuate traditional social prejudices and stigmas (Monteith and Glenn 2016; Executive Office 2016; Partnership on AI 2019; AI HLEG 2019).

### Individual discomfort with the digital economy

There is a major disconnect between corporate and individual perspectives on the use of personal digital data. In a 2018 survey of adults in US, France, UK and Germany, 75% now limit the personal information they share online, only 29% feel that providing data leads to better commercial products and services, and 60% found wearables “creepy” (RSA 2019). In a 2018 survey of adults in the US, only 11% were willing to share health data with technology companies (Day and Zweig 2019). In a 2018 survey of US Facebook users, 74% were unaware Facebook was classifying them, 51% were uncomfortable with this and 27% said the classifications were inaccurate (Hitlin and Rainie 2019). In another 2018 survey in the US, about 60% thought algorithmic decision-making was unfair and that computer programs will always reflect human bias (Smith 2018). In a survey in Germany, users completely rejected sharing mental health information with commercial organizations for developing health recommender systems (Valdez and Ziefle 2019).

### Privacy protection

Patients are very concerned about privacy (Torous et al. 2018), and many are not comfortable providing personal data to clinicians via mobile apps (Dragovic et al. 2018). In the US, most apps fall outside of HIPAA protections, which only apply to traditional healthcare relationships and environments including healthcare providers, insurers and their business associates (Cohen and Mello 2018; Gostin et al. 2018). HIPAA does not cover patient-generated data from apps, wearables or the Internet, which is collected by firms and services that receive, store, combine, analyze and sell the data (Cohen and Mello 2018; Glenn and Monteith 2014b; Monteith and Glenn 2016; Gostin et al. 2018). HIPAA also does not cover the diverse range of non-medical digital data that is routinely included in medically related algorithms (Glenn and Monteith 2014b). HIPAA does apply when data from apps or wearables are sent to the EMR (Hughes 2019; MicroMD 2019). Although the European GDPR requires more robust accountability, some firms circumvent these rules as the data environment becomes increasingly internationalized (Gostin et al. 2018; Vinokur 2019; Scott et al. 2019).

### Privacy policies

For most smartphone apps and wearables, the only protections provided to users are those included in the privacy policy. The requirement for apps to a provide privacy policy is increasing, but varies internationally and by app store. Studies that searched for apps after 2016 found that many lacked a privacy policy, as shown in Table 4. Based on a “notice and consent” model, a privacy policy offers the consumer a take-it-or-leave it choice to agree to the terms (PCAST 2014). Most people just agree without reading privacy policies, which are often unclear, written at a post-secondary level with technical information embedded in legal language (PCAST 2014; Frazee et al. 2016; Robillard et al. 2019). Privacy policies for apps and wearables routinely authorize the sale, transfer, analysis and disclosure of consumer data to third parties (Elvy 2018). For example, an analysis of network traffic and privacy policies of top rated Android apps for prescription medications in Oct-Nov 2017, found that 19 of 24 apps (74%) shared data with 55 unique third parties, who in turn could share with 216 fourth parties (Grundy et al. 2019). The companies receiving this data included large tech companies, Alphabet/Google, Facebook and Oracle, and digital advertising firms, among others. In an analysis of the 36 top-ranked apps for depression and smoking cessation in Jan 2018, 33 of the 36 apps (92%) shared data with a third party, and 29 of the 36 apps (81%) shared data with Google and Facebook (Huckvale et al. 2019). Most privacy policies authorize the sale of personal data in the case of a merger, acquisition or bankruptcy (Elvy 2018, Singer and Merrill 2015). This is pertinent since in the US about 90% of all business startups fail (Patel 2015), including 3 out of 4 venture backed firms (Gage 2012). Additionally, patterns detected in the analysis of large, diverse datasets may allow firms to infer medical conditions and thus undermine the importance of individual consent (Barocas and Nissenbaum 2014).

**Table 4:** Studies of percent of apps searched 2016–2018 that provide a privacy policy

App search	Source	Dates of search	Country of search	Number of apps	Percent with privacy policy (%)	Study
Depression	Google Play, Apple iTunes store	Oct, 2017	US	116	49%	O’Loughlin et al. (2019)
Depression	Google Play, Apple App store	Oct–Nov, 2018	UK	353	74%	Bowie-DaBreo et al. 2019
Depression and smoking cessation	“official Android and iOS marketplaces”	Jan, 2018	US and Australia	36	69%	Huckvale et al. (2019)
“mood” and “track”	Google Play, Apple App store	Jun–Aug, 2016; and Jan–Feb 2017^a^	Canada	319 IOS; 69 Android	18% for IOS; 4% Android^b^	Robillard et al. (2019)
Dementia	Apple iTunes store	Apr–May, 2016	US	72	46%	Rosenfeld et al. (2017)
Specific diseases including mental illnesses	Google Play, Apple iTunes store	July 2018	US	40 each for anxiety, schizophrenia, depression, addiction	85%-anxiety; 50%-schizophrenia; 85%-depression; 70%-addiction	Wisniewski et al. (2019)
Migraine	Google Play, Apple App store, healthline.com	July–Aug, 2017	US	29	76%	Minen et al. (2018)
Mental Health	Google Play	May–Jun, 2017	India	82^c^	35%	Powell et al. (2018)
Increase physical activity	Google Play, Apple iTunes store	Oct, 2016	UK	65	70%	Bondaronek et al. (2018)

Apps in English

^a^Personnel communication

^b^Terms of agreement present for 15% of Apple and 3% of Android

^c^Apps in English of Indian origin

### Security issues for app or wearable data in EMR

As more data from patient apps are sent to the EMR, procedures must be in place to safely handle the data influx. The healthcare industry is a frequent target of all forms of cyberattacks due to extreme vulnerability to disruption in services, and the ability for criminals to monetize the financial and health information contained in medical records (Argaw et al. 2019). These cyber attacks occur worldwide with major incidents reported in the US, UK and Norway (Charette 2018). In the US, data breaches increased between 2010 and 2017 and involved 176.4 million patient medical records (McCoy and Perlis 2018). Ransomware is increasing rapidly (DOJ 2017), with healthcare the most targeted industry (Donovan 2018). Patient portals into EMR have also been compromised (HIPAA Journal 2017). The 2017 US Health Care Industry Cybersecurity Task Force concluded that healthcare cybersecurity is in “critical condition” (HHS 2017). Yet IT spending as a percentage of revenue is much lower in healthcare compared with financial services, another industry that must focus on security (Computer Economics 2019). This is illustrated when considering the IT spending per user, including both employees and non-employees, based on spending at the 25th and 75th percentiles. IT spending per user at the 25th percentile in financial services is $13,772, more than double that of healthcare at the 75th percentile at $6143 (Computer Economics 2019). Lower spending on IT in healthcare than financial services directly impacts security. Even if healthcare and financial services spend the same percentage of the IT budget on security, the dollar amount available in healthcare to obtain expensive IT security products and services is much lower (Computer Economics 2019).

### Individual discomfort with app and wearable data in EMR

Patient security concerns about EMR may impact disclosure of personal information (Agaku et al. 2014), use of patient portals, and use of apps or wearables that send data to an EMR. In US national surveys, about half the people had concerns about privacy and security of medical records (Patel et al. 2016), and 25% of those offered a patient portal would not use due to privacy concerns (Patel and Johnson 2018). In a survey of 12,000 adults, nearly all were concerned that sensitive data such as mental health notes would be shared beyond their chosen provider (Snell 2017). Also in this survey, 89% of patients withheld information, with 93% of these saying the reason was concerns over security of personal financial information. Between 31 and 38% of those surveyed in Canada and the UK said they would postpone seeking care due to privacy concerns (Fair Warning 2011a, b).

## Evolving consumer technology

While we are currently in a technology era dominated by smartphones, this will not last. As predicted by Gordon Bell, leader of the minicomputer revolution at Digital Equipment Corporation, a new class of smaller computers is developed about every decade (Bell 2008). The primary computing platform has evolved from mainframes to minicomputers, to workstations, to PCs, to laptops, to smartphones. For over 50 years, the basis for this change was the doubling of the number of transistors per chip about every 2 years as predicted by Gordon Moore, cofounder of Intel (Moore 2006; Mack 2011). The computing platform will continue to evolve beyond smartphones with each new class being smaller and less expensive (Bell 2008). Indeed, mobile subscription sales are near saturation in the developed world (GSMA 2018a), and smartphone sales have reached a plateau and started to decline (Savov 2019; Swearingen 2018).

In the future, apps will be controlled by voice. Voice interfaces to small, smart wearables including watches, fitness trackers, and ear-pads, are coming soon, as well as to smartphone apps, and a wide range of smart devices and home controls (Koetsier 2019). Miniaturized system components, including sensors and microprocessors with greatly reduced power and energy requirements, and new soft, flexible materials will enable ubiquitous sensing and computing (Lee and Lee 2017; Herbert et al. 2018). Voice assistants marketed today include Amazon Alexa, Google Assistant, Apple Siri, and Microsoft Cortana. The number of voice assistants in use worldwide is estimated to triple to 8 billion by 2023 becoming an $80 billion market (Perez 2019), while smart wearable device sales will double to 233 million units by 2022, becoming a $27 billion market (Lamkin 2018). Consumer electronics shows are dominated by a wide range of voice activated products including TVs, toilets, lightbulbs, ovens, blinds, speakers and showers (Wiggers 2019). The complex issues of privacy, security and commercial involvement, accuracy and efficacy are increasing as consumer devices evolve.

These new voice-activated devices are predicted to replace many of the functions of today’s smartphone, and provide new functions that change how we live. In the words of Google CEO Sundar Pichai “Looking to the future, the next big step will be for the very concept of the “device” to fade away. Over time, the computer itself—whatever its form factor—will be an intelligent assistant helping you through your day” (Google 2016). The pace of adaption of new technologies has greatly accelerated, as it took 85 years for a telephone to become an integral part of life and only 13 years for a smartphone (Irving 2019). People will live with an increasing variety of technology products, and the patterns of usage of current devices such as smartphones will change.

## Limitations of this review

There are limitations to this review. This is not a systematic review of mental health related studies using smartphones apps. Physician perspectives about recommending technology, or the potential impacts of large amounts of patient data in the EMR on physician overload were not discussed. Measures related to improving the usability of apps, and long-term impacts of app use on patient trust were not included. Approaches to rating or recommending apps, legal issues related to app or technology errors, detailed regulatory issues, and technical issues related to privacy, security, and interoperability standards were not discussed. Methodological issues related to the analysis of patient generated data, such as missing values, were not discussed. The requirement to engage in self-tracking outside of medicine, as by employers or insurers, was not discussed. Issues related to anonymization and re-identification of data, and sharing of research data collected from apps, were omitted. Finally, issues related to radiofrequency microwave radiation exposure were not discussed, including cellphone safety limits, emissions when cellphones touch the body, increased absorption rates in children (Gandhi et al. 2012; Gandhi 2019; Fernández et al. 2018; Morris et al. 2015), and potential health effects from long-term exposure (Lin 2018). The article search for this review occurred between February–May, 2019.

## Conclusions

The issues discussed in this paper suggest some recommendations for the future of consumer use of technology in psychiatry.

### Maximize patient choice of technology

The focus of technology in psychiatry should be on automating functions that will allow patient input or contact using many types of consumer technologies, respecting the patient’s lifestyle, budget and skill set. For example, those who can only afford intermittent smartphone service may prefer to receive emails rather than text messages (Alcaraz et al. 2018). It is also important to consider that people of all ages with disabilities, including visual or motor impairments, may prefer to use technology other than a smartphone (Watanabe et al. 2015; Trewin et al. 2013; Bauer et al. 2018a). Patient technologies for medicine, including mental illness, should not exclude those with physical disabilities (Wolbring and Lashewicz 2014).

The era of personalized medicine should recognize that patients use and prefer different types of consumer technologies. A medication reminder system could send text messages to a smartphone or feature phone, email to a laptop, call a standard telephone, or connect with a voice assistant. In a US national sample, the most commonly used health technology in 2018 (by 59%) was to refill prescriptions (Abrams and Korba 2018), which would not require a smartphone. Due to the many limitations discussed above, analyses based on patient data should support many types of technologies, rather than focusing on unregulated, sensor based measurements. A small amount of data could be entered from all commonly used consumer technologies, at a frequency such as daily or weekly.

### Help to improve digital skills

A secondary benefit of recommending the use of technology to those with mental illness is to increase digital skills, and the use of multiple consumer technologies should be encouraged and supported. Some researchers feel that smartphone only access to the Internet is creating a new type of “mobile underclass” with fewer digital skills and more passive online involvement (Napoli and Obar 2014). Studies from diverse countries including The Netherlands and Chile report less information seeking, active participation and variety of Internet use when access is only by smartphone (van Deursen and van Dijk 2019; Correa et al. 2018). In a US study of smartphone users over age 18, 87% of smartphone time was spent on apps and only 13% on the Internet (ComScore 2017b). Programs to help community integration of those with serious mental illness could include training on the safe use of technology.

### Recommendations for the future

Increased understanding of the complex issues surrounding consumer technologies is needed to successfully integrate apps into the practice of psychiatry. New methodologies must be defined and standardized to evaluate the efficacy of apps used for screening or treatment. Regardless of the technology platform, only some patients will use the app. Given the realities of app accuracy, efficacy, privacy, security, and the regulatory environment, and to maximize participation, a variety of technology platforms should be used for data collection rather than focusing on smartphones. Development should also include administrative apps that may increase care participation, and apps that educate about mental illness. App development requires multidisciplinary expertise in medical, legal, consumer, and technical areas, with physicians and patients heavily involved in all phases, and large-scale testing in clinical settings.

Complete security information should be provided to patients before recommending any apps on any technology platform. Training and ongoing support from humans should be available for all recommended apps. Patients should be allowed to choose if they want their app data included in their EMR, shared with anyone other than their psychiatrist, or used in research. Patient data from apps should not be transferred into EMR if insufficient IT resources are available to handle securely, or if unable to accommodate patient choice as to access and use. Finally, investment in programs to increase competence and comfort with technology for those with mental illness should be considered.
